# The complex amino acid diet of *Francisella* in infected macrophages

**DOI:** 10.3389/fcimb.2015.00009

**Published:** 2015-02-06

**Authors:** Monique Barel, Elodie Ramond, Gael Gesbert, Alain Charbit

**Affiliations:** ^1^Université Paris Descartes, Sorbonne Paris CitéParis, France; ^2^INSERM U1151 - Centre National de la Recherche Scientifique UMR 8253, Institut Necker-Enfants MaladesParis, France

**Keywords:** nutrition, intracellular pathogen, amino acid uptake, phagosomal escape, *Francisella*

## Abstract

*Francisella tularensis*, the agent of the zoonotic disease tularemia, is a highly infectious bacterium for a large number of animal species and can be transmitted to humans by various means. The bacterium is able to infect a variety of cell types but replicates in mammalian hosts mainly in the cytosol of infected macrophages. In order to resist the stressful and nutrient-restricted intracellular environments, it encounters during its systemic dissemination, *Francisella* has developed dedicated stress resistance mechanisms and adapted its metabolic and nutritional needs. Recent data form our laboratory and from several other groups have shown that *Francisella* simultaneously relies on multiple host amino acid sources during its intracellular life cycle. This review will summarize how intracellular *Francisella* use different amino acid sources, and their role in phagosomal escape and/or cytosolic multiplication and systemic dissemination. We will first summarize the data that we have obtained on two amino acid transporters involved in *Francisella* phagosomal escape and cytosolic multiplication i.e., the glutamate transporter GadC and the asparagine transporter AnsP, respectively. The specific contribution of glutamate and asparagine to the physiology of the bacterium will be evoked. Then, we will discuss how *Francisella* has adapted to obtain and utilize host amino acid resources, and notably the contribution of host transporters and autophagy process in the establishment of a nutrient-replete intracellular niche.

## Introduction

*Francisella tularensis* is a small Gram-negative bacillus, aerobic, non-spore-forming, and non-motile. This facultative intracellular pathogen is the causative agent of the zoonotic disease tularemia in a large number of animal species. This highly infectious bacterial pathogen can be transmitted to humans in numerous ways (Sjostedt, 2011), including direct contact with sick animals, inhalation, ingestion of contaminated water or food, or by bites from ticks, mosquitoes or flies. Four different subspecies (subsp.) of *F. tularensis* that differ in virulence and geographic distribution exist, designated subsps. *tularensis, holarctica, mediasiatica*, and *novicida*, respectively. The most virulent subspecies *tularensis* is considered a potential Class A agent in bioterrorism by the Centers for Disease Control (CDC) in the United States (Oyston et al., 2004; Keim et al., 2007). *F. tularensis* subsp. *novicida* (*F. novicida*) is rarely pathogenic to non-immunocompromized humans but is fully virulent for mice and is therefore widely used as a model to study *Francisella* intracellular parasitism.

*F. novicida* has the capacity to evade host defenses and to replicate to high numbers within the cytosol of eukaryotic cells (Jones et al., 2012). The bacterium is able to replicate inside a variety of cells, and in particular in macrophages. After a transient passage through a phagosomal compartment, bacteria are released within 30–60 min in the host cell cytosol where they undergo several rounds of active replication (Celli and Zahrt, 2013). At least 20% of the genome participates to some extent to *Francisella* virulence (Meibom and Charbit, 2010b), including an important proportion of genes related to metabolic and nutritional functions. However, understanding the relationship between nutrition and the *in vivo* life cycle of *Francisella* is still poorly understood.

*Francisella* is predicted to possess numerous nutrient uptake systems to capture its necessary host-derived nutrients, some of which are probably available in limiting concentrations. We will review here our recent findings regarding two *Francisella* amino acid acquisition systems and their importance in the physiology and intracellular life cycle of *Francisella*. We will also discuss the major host responses, identified to date, triggered upon *Francisella* infection that contribute to fuel the cytosolic compartment.

## *Francisella* amino acid transporters play a critical role in intracellular multiplication

We have previously shown that *Francisella* used the cysteine-containing tripeptide glutathione (GSH) as a source of cysteine, to replicate in infected macrophages (Alkhuder et al., 2009), thus suggesting that this bacterium has evolved by exploiting the natural abundance of GSH in the host cytosol to compensate its natural auxotrophy for cysteine. More recently, we decided to evaluate the role of amino acid transport systems in the capacity of *Francisella* to thrive intracellularly.

As mentioned in an earlier review (Meibom and Charbit, 2010a), most of the transport systems encoded by *Francisella* genomes are secondary carriers, corresponding to up to 75% of the predicted transport proteins in *F. tularensis*. These active transport systems use the electrochemical gradient to translocate elements across the bacterial cytoplasmic membrane, either by symport (two components moving in the same direction) or by antiport (two molecules moving in opposite direction). Secondary transporters of *Francisella* encompass several major families such as: (i) the major facilitator superfamily (MFS), involved in various functions, including drug efflux, sugar and amino acid uptake (Meibom and Charbit, 2010a), and comprising 31 proteins; (ii) the amino acid/polyamine/organocation (APC) subfamily, comprising 11 proteins; (iii) the subfamily of permeases for hydroxy aromatic amino acids (HAAAP), comprising 7 proteins; and (iv) the oligopeptide dependent proton (POT) subfamily of transporters, comprizing 8 proteins. Remarkably, 52 proteins involved in secondary active transport were found in earlier *in vitro* or *in vivo* genetic screens as being involved in *Francisella* pathogenicity (Qin and Mann, 2006; Tempel et al., 2006; Maier et al., 2007; Su et al., 2007; Weiss et al., 2007; Kraemer et al., 2009; Asare and Abu Kwaik, 2010; Asare et al., 2010; Moule et al., 2010; Peng and Monack, 2010), among which 61% of the MFS transporters.

We will discuss below the data that we have obtained very recently on GadC and AnsP, two secondary transporters being specifically involved in phagosomal escape and cytosolic multiplication, respectively.

### The APC family members

*F. tularensis* genomes encode 11 predicted members of the amino acid-polyamine-organocation (APC) superfamily transporters, specifically involved in amino acids exchange w/o ions. Remarkably, 8 of the 11 APC members have been identified at least once in earlier genetic studies (*in vitro* or *in vivo*). The functional role of one of them, the glutamate transporter GadC, was elucidated in our laboratory (Ramond et al., 2014) and its essential contribution to *Francisella* phagosomal escape was demonstrated.

### Glutamate uptake is critical in the phagosome

The GadC-encoding gene (*FTN_0571* in *F. novicida*) had been identified in four studies as an essential actor in *F. tularensis* subsp. *holarctica* (Maier et al., 2007) and *F. novicida* (Weiss et al., 2007; Kraemer et al., 2009; Peng and Monack, 2010) virulence. Inactivation of the gene *FTN_0571* in *F. novicida* confirmed its implication in phagocytic cells multiplication such as J774.1, THP-1 and bone marrow-derived macrophages (BMMs) but also in virulence (in BALB/c mice). We showed that this transporter was essential for proper bacterial phagosomal escape. Furthermore, our analyses revealed that GadC contributed to the resistance to reactive oxygen species (ROS) generated in the phagosome by importing glutamate. Remarkably, the intracellular multiplication defect, as well as attenuated virulence in mice of the Δ*gadC* mutant, was fully or partially reversed in phox-KO BMMs and phox-KO mice, respectively. Altogether, our data were consistent with the notion that imported glutamate was used by *F. tularensis* to fuel the tricarboxylic acid cycle (TCA) in the phagosomal compartment, revealing thus a new role of glutamate utilization in oxidative stress defense. Of note, GadC was previously shown to play a specific role in acid resistance challenge, especially in *E. coli* (Castanie-Cornet et al., 1999) and *L. monocytogenes* (Cotter et al., 2001), two enteropathogenic bacteria that must resist a severe acid stress during their natural infectious route. The role of GadC in oxidative stress resistance in *F. tularensis*, which is not an enteropathogen, may thus, illustrate an adaptation of bacterium to the environments it encounters during its *in vivo* life cycle.

### The MFS transporters

The importance of the MFS family in the virulence of intracellular bacteria has been demonstrated for the first time in *Legionella pneumophila*. Indeed, a threonine transporter (called PhtA for Phagosomal Transporter A) has been identified as unable to differentiate into replicative form in macrophages and as expressing an early stage of the factors leading to the transmissive form in culture medium (Sauer et al., 2005). The *L. pneumophila* genome encodes 10 additional PhtA paralogues (Sauer et al., 2005), some of which are also required during intracellular replication (Fonseca and Swanson, 2014). PhtJ is required for acquisition of valine and PhtC and PhtD were very recently shown to contribute in protecting *L. pneumophila* from dTMP starvation (Fonseca et al., 2014).

Strikingly, Pht transporters constitute a sub-family of MFS transporters found exclusively in intracellular pathogens and specifically in Alpha and Gamma-proteobacteria (including *L. pneumophila*, *Coxiella burnetii*, *Rickettsiella grylli*, *Francisella tularensis*, *Wolbachia*, *Anaplasma*, *Ehrlichia*, *Protochlamidia amoebophila*, *Prochlorococcus marinus*, and *Zymomonas mobilis* (Chen et al., 2008).

This led us to address in priority the role of the Pht subfamily members in *Francisella* pathogenesis. The six Pht transporters identified in *Francisella* are highly conserved in the different sub-species (>95% amino acid identity). Each of these transporters has been found to contribute to *F. tularensis* virulence (Qin and Mann, 2006; Weiss et al., 2007; Kraemer et al., 2009; Asare and Abu Kwaik, 2010; Asare and Kwaik, 2010; Moule et al., 2010; Peng and Monack, 2010; Llewellyn et al., 2011).

The functional role of two Pht transporters of *F. tularensis* was very recently elucidated in our laboratory (Gesbert et al., 2014, 2015). We will focus below on one of them, the asparagine transporter AnsP that contributes exclusively to the cytosolic multiplication of the pathogen.

### Asparagine uptake is critical for cytosolic multiplication

Inactivation of the *ansP* gene in *F. tularensis* subsp *tularensis* SCHU S4, *F. tularensis* subsp *holarctica* LVS or *F. tularensis* subsp *novicida* U112, causes a decrease in intracellular multiplication in different cellular models such as HepG2 human hepatocytes, primary murine macrophages and murine macrophages J774 (Qin and Mann, 2006; Marohn et al., 2012; Gesbert et al., 2014). *In vivo*, the inactivation of the *ansP* gene causes a significant decrease in virulence of *F. tularensis* subsp *novicida* U112 in *Drosophila* (Asare and Abu Kwaik, 2010); and *F. tularensis* subsp *holarctica* LVS in BALB/c mice (Marohn et al., 2012).

We showed that the impaired intracellular growth and the *F. novicida* Δ*ansP* mutant could be abrogated upon supplementation with an excess of asparagine: (i) in a free form or as a dipeptide, in cell culture models; (ii) by intraperitoneal injection of asparagine, in infected mice (Gesbert et al., 2014). Thus, whereas the bacterium is prototrophic for asparagine during growth in synthetic medium, it becomes phenotypically auxotrophic for asparagine in the cytosol of infected cells. A similar phenomenon has been also described in *M. tuberculosis*. This bacterium, which is prototrophic in culture for the 20 amino acids, possesses two transporters, one for aspartate and one for asparagine, which are important for intracellular mycobacterial survival and multiplication (Gouzy et al., 2013, 2014). Thus, the existence of a predicted active biosynthetic pathway does not preclude the requirement of an efficient transport system to support intracellular multiplication. These results support the generally accepted assumption that nutrient uptake is favored to synthesis when the nutrient is available in the external medium. We also demonstrated that the AnsP-mediated capture of asparagine by *Francisella* had no impact on phagosomal escape but became critical as soon as the bacterium reached the host cytoplasm (Gesbert et al., 2014). The data obtained further suggested that cytosolic bacteria use asparagine mainly as a building block for protein synthesis. However, additional metabolic or regulatory functions of asparagine cannot be excluded.

**Figure 1:**
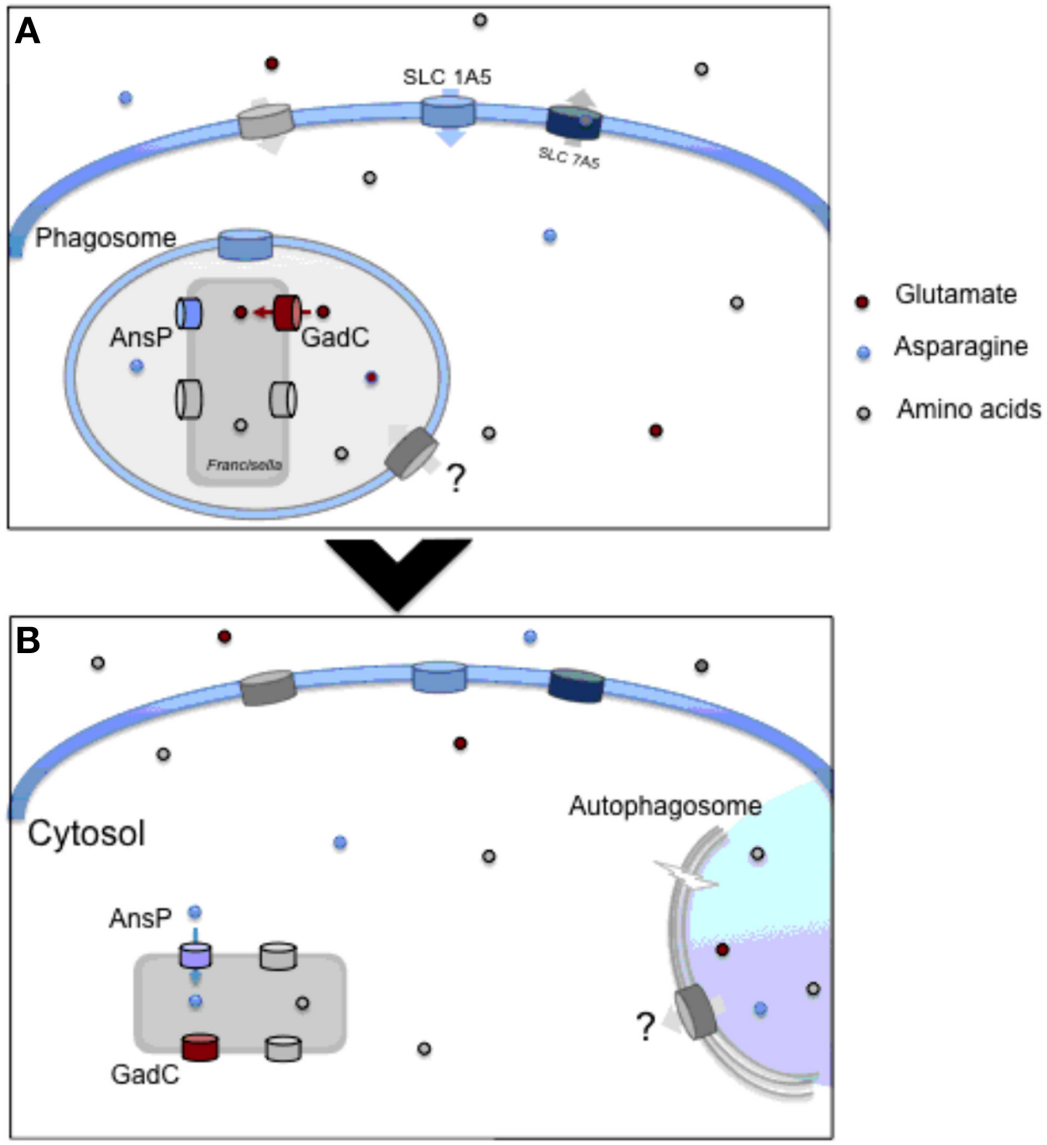
**Amino acid utilzation by intracellular *Francisella***. **(A)** Upon entry into macrophages, *Francisella* transiently resides in a phagosomal compartment. In this compartment, the glutamate permease GadC helps the bacterium to resist the oxidative stress generated by the NADPH oxidase. This transporter is critically required for proper bacterial phagosomal escape. Concomitantly, the synthesis of the host neutral amino acid transporter SLC1A5 is rapidly up-regulated, favoring the entry of amino acids in the infected host cell. **(B)** Once in the host cytosol, bacteria start their active replication and therefore require the supply of numerous additional host-derived nutrients. The AnsP permease contributes to bacterial multiplication by providing asparagine for protein synthesis. The ATG-5-independent autophagy contributes to the delivery of amino acids, enriching the cytosolic bacterial diet. At both stages, other amino acid transporters contribute to the proper feeding of the bacterium.

It is likely that other members of the MFS and APC families (and possibly of other families) yet to be discovered, may participate to the intracellular life cycle of *Francisella* (Figure 1).

## Host-derived amino acid sources

The host cytosol, previously considered as a safe nutrient-replete (Ray et al., 2009), is now established as a life-threatening nutrient-deprived environment for the invading bacteria (Abu Kwaik and Bumann, 2013). A permanent war takes place between the host and the pathogen. Indeed, intracellular pathogens try to deprive nutrients from their host, while the host cell tries to deprive the invading bacteria of nutrients. This phenomenon is generally designated “nutritional immunity” (Barel and Charbit, 2013).

### Host amino acid transporters

Involvement of a eukaryotic glutamine transporter SLC1A5 has been recently described during *F. tularensis* LVS infection (Barel et al., 2012). *F. tularensis* LVS induces up-regulation of this transporter both at the mRNA and the protein level. Furthermore, the bacteria infection induces the deglycosylation of this aminoacid transporter. This deglycosylation process increases with the time of infection and is correlated with the increase in SLC1A5 expression. The *IglC* mutant, that does not egress from the phagosome and does not muliply in the cytosol, does not induce this deglycosylation. Therefore, this deglycosylation is induced only by bacteria, which are able to evade the phagosome and multiply into the cytosol.

In the same time, SLC7A5 mRNA and protein expression is down-regulated. SLC7A5 is a partner of SLC1A5 as they work along to equilibrate the cytoplasmic amino acid pool (Fuchs and Bode, 2005) and especially of glutamine (Gln). The differential effect of *F. tularensis* LVS infection on SLC1A5/SLC7A5 expression could therefore induce an increase in intracellular concentration of glutamine. This nutrient plays an important role in regulating gene expression, protein turnover, anti-oxidative function, nutrient metabolism, immunity, and acid-base balance. Use of glutamine by human cells, for controlling *Francisella* infection remains to be studied. We have found (Barel and Charbit, 2013) that addition of glutamine increased the ability of *F. tularensis* LVS to multiply in the cytosol of infected THP-1 cells. Although classified as “non-essential,” glutamine appears essential for the viability and growth of cells maintained in tissue culture by serving notably as a metabolic precursor in several biosynthetic pathways, or directly for protein synthesis (Neu et al., 1996). Glutamine deprivation mediated through *Helicobacter bilis* γ-Glutamyl-transpeptidase has been found to be responsible for induction of inflammatory disorders in epithelial cells (Javed et al., 2013).

Of note, SLC1A5 gene is activated by insulin, through SGK1, SGK3, and PKB kinase activation, which stimulates amino acid absorption (Palmada et al., 2005). In good relationship, we indeed found that addition of insulin also increased *F. tularensis* LVS intracellular multiplication (Barel et al., 2012). Glutamine may be converted into glutamate that in turn, can be used either by the bacteria or by the human cells to provide metabolic advantages and prevent *Francisella* infection. As discussed above, the glutamate transporter of *Francisella* (GadC) has been shown to be critical for oxidative stress defense in the phagosome (Ramond et al., 2014).

The deglycosylation of SLC1A5 seems to be a general mechanism induced by *F. tularensis* infection, as deglycosylation of another highly glycosylated protein (CD147) was also observed. Protein deglycosylation triggered by intracellular *Francisella* may therefore serve also as a nutrient source. The glycan produced could serve in networks regulating cellular involvement directed toward survival of intracellular bacteria.

### Autophagy

Utilization of the autophagy pathway has recently been shown to constitute another mechanism of bacterial adaptation, contributing to the survival and nutrition of intracellular *Francisella* (Steele et al., 2013). Interestingly, Kawula and co-worker have found that intracellular *F. tularensis* subsp *tularensis* SCHU S4 relied on ATG5-independent autophagy for multiplication. Supplying excess pyruvate or amino acids suppressed the bacterial growth defect in autophagy-deficient cells, suggesting a direct role of this process in supplying amino acid. However, *F. tularensis* subsp *holarctica* LVS intracellular growth appeared to be significantly impaired in ATG5^−/−^ macrophages. It is thus likely that this subspecies may use both ATG5-dependent and ATG5-independent autophagy processes to support efficient intracellular growth. Altogether, these data indicate that wild-type intracellular *F. tularensis* generally avoid engulfment by classical autophagosomes but may use autophagy to fuel cytosolic bacterial replication with amino acid. It is possible that other nutrients are provided to intracellular bacteria by this process (such as carbohydrates or lipids).

These two hosts responses triggered upon infection reflect the capacity of intracellular *Francisella* to utilize the eukaryotic machinery for producing amino acids and glycans residues, which may serve for its own survival.

## Concluding remarks

We have shown that two *Francisella* transporters, involved in the uptake of asparagine and glutamate, played a critical role in the bacterial intracellular life cycle. Remarkably, these two amino acids are two non-essential amino acids, thus implying that prototrophy in broth does not necessarily predict independence toward host amino acid source(s) during infection. Comparison of predicted nutrient utilization and biosynthetic pathways of a series of mammalian pathogens support the notion that most pathogens share the capability to simultaneously utilize multiple nitrogen and carbon sources (Abu Kwaik and Bumann, 2013; Steeb et al., 2013). Our ongoing studies indicate that *Francisella* also relies on several other host-derived amino acid sources to multiply inside infected macrophages (Gesbert et al., 2015, unpublished).

Other host nutrient sources are likely to be involved in *Francisella* intracellular adaptation. For example, Weiss and co-workers have shown that biotin biosynthesis was required in the *Francisella* phagosome to promote rapid bacterial escape (Napier et al., 2012), providing the first example of a metabolic requirement of *Francisella* in this compartment. The importance of biotin biogenesis in phagosomal escape may suggest that biotin is limiting in this compartment. If so, sequestration of biotin could constitute a form of nutritional immunity by the host innate immune system. These hypothesis remains to be experimentally confirmed. Iron is also limiting in the phagosome and numerous host factors play a critical role in the control of iron sequestration upon microbial infection. *F. tularensis* subsp *holarctica* LVS infection has been shown to trigger a significant up-regulation of transferrin receptor expression, ultimately leading to increased intracellular iron availability. However, the host combats this iron influx by expelling iron out of the phagosomes as well as by increasing the expression ferroportin, which shuttles iron from the cytosol out of the cell. In turn, to reduce ferroortin activity, *F. tularensis* subsp *holarctica* LVS infection induces the production of hepcidin that binds to ferroportin on the host cell surface, leading to its subsequent degradation (Pan et al., 2010; Jones et al., 2012). As a result, *Francisella* is able to maintain a sufficient intracellular iron pool.

### Conflict of interest statement

The authors declare that the research was conducted in the absence of any commercial or financial relationships that could be construed as a potential conflict of interest.
